# An Excitatory/Inhibitory Switch From Asymmetric Sensory Neurons Defines Postsynaptic Tuning for a Rapid Response to NaCl in *Caenorhabditis elegans*

**DOI:** 10.3389/fnmol.2018.00484

**Published:** 2019-01-09

**Authors:** Masahiro Kuramochi, Motomichi Doi

**Affiliations:** Molecular Neurobiology Research Group and DAILAB, Biomedical Research Institute, National Institute of Advance Industrial Science and Technology (AIST), Tsukuba, Japan

**Keywords:** synapse integration, *C. elegans*, salt-chemotaxis behavior, Ca^2+^ imaging, glutamate

## Abstract

The neural networks that regulate animal behaviors are encoded in terms of neuronal excitation and inhibition at the synapse. However, how the temporal activity of neural circuits is dynamically and precisely characterized by each signaling interaction *via* excitatory or inhibitory synapses, and how both synaptic patterns are organized to achieve fine regulation of circuit activities is unclear. Here, we show that in *Caenorhabditis elegans*, the excitatory/inhibitory switch from asymmetric sensory neurons (ASEL/R) following changes in NaCl concentration is required for a rapid and fine response in postsynaptic interneurons (AIBs). We found that glutamate released by the ASEL neuron inhibits AIBs *via* a glutamate-gated chloride channel localized at the distal region of AIB neurites. Conversely, glutamate released by the ASER neuron activates AIBs *via* an AMPA-type ionotropic receptor and a G-protein-coupled metabotropic glutamate receptor. Interestingly, these excitatory receptors are mainly distributed at the proximal regions of the neurite. Our results suggest that these convergent synaptic patterns can tune and regulate the proper behavioral response to environmental changes in NaCl.

## Introduction

Neuronal circuit activity is modulated by a balanced and well-organized combination of excitatory and inhibitory signals. The circuit dynamics are temporally and spatially regulated by rate of synaptic transmission and are intrinsic for the brain functions that regulate numerous animal behaviors. Such behavioral responses to sensory stimuli are dynamic and finely tuned largely as a result of the interactions between excitatory and inhibitory signals. In the vertebrate visual system, the responses of ON and OFF bipolar neurons are finely tuned by photoreceptor neurons *via* both excitatory and inhibitory synapses, and these bipolar neurons then transmit visual information to upper layer ganglion cells (Schiller et al., 1986). An information stream to discriminate between brightness and contrast in the retina has also been proposed to be strictly regulated by excitatory and inhibitory signals (Molnar et al., 2009). In an analogous fashion, even robotic control systems integrate positive and negative signals to finely regulate dynamic operations with quick and precise movements (Brooks, 1986). This strategy of using positive and negative signaling to finely regulate systems is thus widely adopted from machines to multicellular organisms. However, how each positive (excitatory) and negative (inhibitory) synapse in the animal brain contributes to the overall temporal activity of circuits, and how both types of synapse are organized in a certain circuit to regulate activity at the single neuron level is unclear.

The nematode *Caenorhabditis elegans* is an accessible and valuable model to characterize neural circuit dynamics at the cellular network level and the subcellular synaptic level. As such, the synaptic connectivity of the whole *C. elegans* nervous system has been identified (White et al., 1986). Worms can execute various behaviors, from simple withdrawal reflexes, thermotaxis and chemotaxis, to more the complex associative learning and memory, despite having a small nervous system of only 302 neurons (Mori and Ohshima, 1995; Wicks and Rankin, 1995; Nuttley et al., 2002; Kunitomo et al., 2013). A combination of genetic manipulation, laser ablation, calcium imaging and optogenetic techniques have been used to map the neural circuits regulating these behaviors in worms, and determine the neuronal dynamics underlying these behaviors at the cellular level. In particular, calcium imaging is often used to monitor brain-wide activity at the cellular resolution level (Prevedel et al., 2014; Kato et al., 2015).

The salt-chemotaxis behavior exhibited by *C. elegans* is regulated by a pair of chemosensory ASE neurons (ASEL and ASER) and their postsynaptic interneurons. ASEL and ASER both detect changes in NaCl concentration, yet produce opposite responses to the NaCl change. ASEL is activated by increases in NaCl concentration, whereas ASER is activated by decreases in NaCl concentration (Suzuki et al., 2008). Furthermore, these neurons have distinct functions in mediating chemotaxis behavior: ASEL activation promotes forward run probability, whereas ASER activation promotes turn probability (Suzuki et al., 2008; Thiele et al., 2009). Despite these asymmetric properties, both neurons probably use glutamate as a neurotransmitter, based on the expression of the vesicular glutamate transporter EAT-4 (Serrano-Saiz et al., 2013) and connect to several of the same interneurons, including AIB and AIY interneurons (White et al., 1986). The AIB interneurons regulate the reversal/turn behavior and communicate with both ASEL and R sensory neurons *via* chemical synapses. These findings provoke an intriguing question as to what synaptic signaling mechanism exists between the same interneurons that can generate opposing behavioral outputs as a result of asymmetric responses in the receiving sensory neuron using the same transmitter (glutamate).

Kunitomo et al. (2013) reported that AIB interneurons are activated by the decrease of NaCl concentration, possibly dependent on the synchronous activation of the ASER neuron. On the other hand, these AIB are inhibited when ASEL is activated (Wang et al., 2017). These findings suggest that switch from excitatory by ASER to inhibitory by ASEL or vice versa may precisely regulate the AIB activity and affect forward and backward locomotion in salt chemotaxis. However, it is not clear whether such excitatory/inhibitory switch is really involved in the ASE-AIB synaptic circuit, and how this switch is regulated in molecular level. Here we show that in the *C. elegans* salt-chemotaxis circuit, the excitatory/inhibitory switch from asymmetric sensory neurons defines postsynaptic tuning and smooth transition of activity to changes in NaCl concentration. We also show that glutamate released by the ASEL inhibits postsynaptic AIBs through a glutamate-gated chloride channel and an unidentified receptor, whereas glutamate released by the ASER activates AIBs *via* AMPA-type ionotropic and G-protein-coupled metabotropic glutamate receptors. Furthermore, each excitatory or inhibitory synapse is located on distinct regions of the postsynaptic AIB neurite. These results suggest that excitatory/inhibitory signaling from asymmetric sensory neurons is integral to the salt-chemotaxis neural circuit to achieve rapid and fine responses in postsynaptic neurons for suitable behavioral decisions.

## Materials and Methods

### Strains

Worms were cultivated on standard NGM agar plates seeded with *E. coli* OP50 at room temperature (~22°C). The Bristol N2 was used as wild-type strain, and other mutant strains and transgenic strains used in this study are listed in Supplementary Table S1.

### Molecular Biology and Transgenic Animals

Standard methods for molecular biology were used to construct plasmid DNAs. For the expression of the calcium indicator protein G-GECO1.2 (a kind gift from Takeshi Ishihara) or GCaMP6 (kind gift from Junichi Nakai), each coding sequence was inserted between the AgeI and EcoRI sites of the pPD95.79 vector (kind gift from Andy Fire). Then, the promoter region for cell-specific expression of the cDNAs was inserted between the SphI and XmaI sites of the resulting pPD95.79/G-GECO1.2 or pPD95.79/GCaMP6 plasmids. We used the following promoters for cell-specific expression: *gcy-5* for ASER, *gcy-7* for ASEL, and *npr-9* for AIB. To generate plasmid DNAs for cell-specific UNC-13 expression, the full-length *unc-13* cDNA fragment was amplified by overlapping PCR fusion using the following primers:

- 5′CCGGGATGCCACGCCGACGGAAACGAAA3′
- 5′GTGTCCTTCGTTTGGTCTTTCCAACTTGAG3′
- 5′CTCAAGTTGGAAAGACCAAACGAAGGACAC3′
- 5′CAGGCGTCTTGCATCGTTTCTTTTG3′
- 5′CAAAAGAAACGATGCAAGACGCCTG3′
- 5′GCATTCGGCAGTTGTTTCAATAGAGCC3′
- 5′GGCTCTATTGAAACAACTGCCGAATGC3′.

The resulting full-length UNC-13 cDNA fragment was inserted between the XmaI and KpnI sites of the pPD95.79 Vector. Then, each promoter sequence for ASEL or ASER was inserted between the SphI and XmaI sites of the pPD95.79/UNC-13 plasmid DNA. To generate a *tetanus toxin* expression plasmid, the *TeTx::mCherry* fragment from *Pttx-3::TeTx::mCherry* (a kind gift from Sreekanth Chalasani) was exchanged with the GCaMP6 sequence of the P*gcy-5*::GCaMP6 plasmid DNA. For the expression of the glutamate-gated chloride channel *glc-3*, the *glc-3* cDNA fragment was amplified by PCR fusion using the following primers:

- 5′ATGCGGATCCATGAGTCTCCGTTCACTTCTCAAT3′ and
- 5′TTCTACCGGTACCTTGGCTTCCGGTGCGTGATATTGT3′.

The resulting full-length *glc-3* cDNA was inserted between the BamHI and KpnI sites of the pPD95.79/Venus Vector. Then, the *npr-9* promoter region was inserted between the SphI and BamHI sites of the pPD95.79/*glc-3* plasmid DNA. For the expression of an AMPA-type ionotropic glutamate receptor *glr-1*, the *glr-1::GFP* fragment was excised by KpnI and EcoRV digestion from the *Pttx-3::glr-1::GFP* plasmid (a kind gift from Takaaki Hirotsu), and then inserted into the *Pgcy-7*::R-GECO1 plasmid DNA. Finally, the *npr-9* promoter region was inserted between the KpnI and ApaI sites. For the rescue experiments of *glc-3* or *glr-1* mutants, Venus or GFP sequence was removed from each expression plasmid DNA by using in-Fusion reaction (Takara), respectively.

For the generation of transgenic animals, the resulting plasmid DNAs were injected into N2 (Bristol) or mutant animals using a standard microinjection method (Mello et al., 1991). Details of the strains used in this study are listed in Supplementary Table S1.

### Calcium Imaging

Calcium imaging was performed as described previously (Kuramochi and Doi, 2017). Adult transgenic worms were used for imaging. Worms were immobilized in a microfluidic device fabricated from polydimethylsiloxane (PDMS; Chronis et al., 2007). The microfluidic device was set on an inverted fluorescent microscope (Olympus IX71), and time-lapse images were captured (10 frames/s) using an ORCA-Flash 4.0 CCD camera (Hamamatsu Photonics) controlled by HCImage software (Hamamatsu Photonics). Recordings started within 5 min after removal from food. The following buffers for calcium imaging were used: 5 mM KPO_4_ (pH 6.0), 1 mM CaCl_2_, 1 mM MgSO_4_, including 0 or 50 mM NaCl for the stimulation. All the buffers were adjusted to 350 mOsmol/L H_2_O with glycerol (Oda et al., 2011). The patterns of salt stimulation were automated using the Perfusion Valve Controller System VC-6M (Warner Instruments) and Arduino microcontroller to control solenoid valves (Arduino SRL) with a pre-generated sequence. We used Δ*F*/*F*_0_ to indicate fluorescence intensity change. *F*_0_ was defined as the average fluorescence in a 5 s window before stimulation. After background subtraction, the total fluorescence intensity was measured from individual regions of interest (ROIs) in each neuron. An animal was imaged twice, with a 30 s interval between the first and second observation. For the comparison of Δ*F*/*F*_0_ among genotypes, “response (Δ*F*/*F*_0_)” was calculated using the average fluorescence in a 20 s window when NaCl concentration is decreased or in a 10 s window when NaCl concentration is increased.

### Confocal Microscopy and Synapse Observation

L4 larvae were mounted on a 1.5% agarose pad with 20 mM sodium azide in M9 solution for anesthesia. Images were acquired on an inverted confocal microscope (Nikon A1, Nikon) with a 60× objective lens, and were analyzed by NIS-Elements C/NIS-Elements C-ER and ImageJ software, respectively. The Z-stack image was acquired from the whole animals expressing each fluorescent fusion proteins. Co-localization between GLR-1 or GLC-3 GFP fusion proteins and a presynaptic mCherry::RAB-3 fusion protein was quantified by counting the number of GFP pixels overlapping with mCherry signal in a single *z*-axis flame. The number of pixels in each frame of Z-scan was averaged in each animal and used for quantitative analyses.

### Statistical Methods

All data, except for the ASE activity, did not show gaussian distribution based on the Shapiro–Wilk test. Thus, a non-parametric Wilcoxon rank sum test was used to evaluate the median difference in calcium response.

## Results

### Calcium Responses in Presynaptic Sensory ASE and Postsynaptic AIB Neurons Following Changes in NaCl Concentration

**Figure 1:**
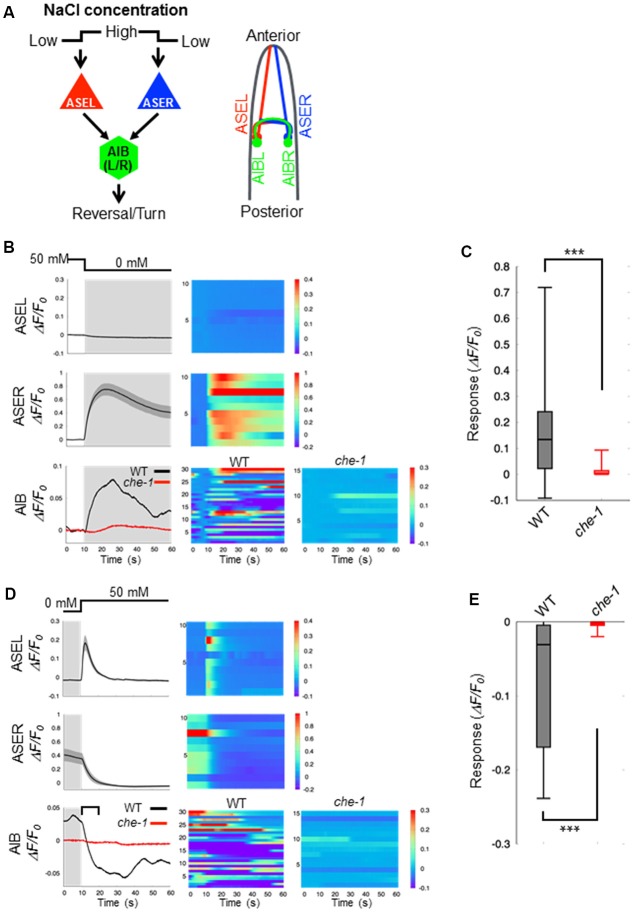
Calcium dynamics in ASEL, ASER and AIB neurons in response to changes in NaCl concentration. **(A)** Simplified synaptic connections between ASE sensory neurons (ASEL and ASER) and AIB interneurons. Both ASE neurons respond to changes in NaCl concentration and connect to several interneurons, including AIB. AIB neurons trigger reversal and turning behaviors. **(B)** Calcium dynamics of ASEL, ASER and AIB neurons in response to a downstep in NaCl concentration from 50 mM to 0 mM. Averaged calcium responses to changes in NaCl concentration (left) and heatmap traces of individual worms (right). The black line indicates the calcium responses in wild-type animals (*n* = 10), and the red line indicates the calcium responses in *che-1(p679)* mutants (*n* ≧ 15). Shaded areas indicate the SEM. **(C)** Quantitative analysis of the maximum calcium responses in AIB neurons during a downstep in NaCl concentration in the wild type (gray) and *che-1* mutants (red). The error bars indicate the SEM; ***p < 0.001; Wilcoxon rank sum test. **(D)** Calcium dynamics of ASEL, ASER and AIB neurons in response to an upstep in NaCl concentration from 0 mM to 50 mM. Averaged calcium responses to changes in NaCl concentration (left) and heatmap traces of individual worms (right). The black line indicates the calcium responses in the wild-type worms (*n* = 10), and the red line indicates the calcium responses in *che-1(p679)* mutants (*n* ≧ 15). The shaded areas indicate SEM. **(E)** Quantitative analysis of the maximum calcium responses in AIB neurons during the first 10 s (blanket in **D**) after an upstep in NaCl concentration in wild type (gray) and *che-1(p679)* mutants (red). The error bars indicate the SEM; ***p < 0.001; Wilcoxon rank sum test.

*C. elegans* detect NaCl concentration gradients *via* the ASEL and ASER sensory neurons and move to a preferential NaCl condition by using several behavioral strategies (Pierce-Shimomura et al., 1999; Iino and Yoshida, 2009). Both ASEL/R have synaptic connections to downstream first-layer interneurons including AIB neurons (Figure 1A). To understand the role of presynaptic excitatory/inhibitory switch in postsynaptic responses of AIB neurons, we recorded fluorescent changes of the genetically-encoded calcium indicator G-GECO1.2 (Zhao et al., 2011) that is specifically expressed in either pre- or postsynaptic neurons. Consistent with previous reports (Suzuki et al., 2008), the ASEL neuron did not show any response to a downstep in NaCl concentration (from 50 mM to 0 mM NaCl), whereas the ASER neuron showed a large, long-lasting response to the downstep (Figure 1B). Conversely, the ASEL neuron showed a fast calcium response to an upstep in NaCl (0 mM to 50 mM), and its response immediately decayed to the steady state level. The ASER calcium response decayed immediately after an upstep in NaCl concentration (Figure 1D). The AIB neurons showed a similar response pattern as the ASER: their responses rose slowly after a downstep in NaCl concentration, and after the peak level, slowly decayed during the exposure to 0 mM NaCl (Figure 1B). In response to an upstep in NaCl concentration, the AIB calcium level immediately decayed to the steady-state level (Figure 1D). Thus, as shown in previous report (Kunitomo et al., 2013; Wang et al., 2017), AIB neurons show similar neuronal responses to both up and down changes in NaCl concentration as the ASER neuron.

Sensory neurons, including AFD, ASE, ASH, ASJ, AWB, and AWC, are thought to function as salt-sensing neurons (Thiele et al., 2009; Zaslaver et al., 2015), and AIB interneurons receive synaptic inputs from many sensory neurons. To examine whether the AIB neuronal responses to changes in NaCl concentration are regulated by ASE neuronal activity specifically, we recorded the AIB response in *che-1(p679)* mutant worms that specifically lacks ASE neurons (Chang et al., 2003; Uchida et al., 2003). Here we found that the AIB neurons in *che-1* mutants showed no significant calcium response to either a downstep or upstep in NaCl concentration (Figures 1B–E). These results indicate that ASE neurons strongly affect AIB activity in response to NaCl concentration changes; other sensory neurons probably have a weak or no effect on AIB activity as they could not compensate for a loss of ASE neurons.

### ASEL Inhibits AIB Activity Whereas ASER Stimulates AIB Activity

**Figure 2:**
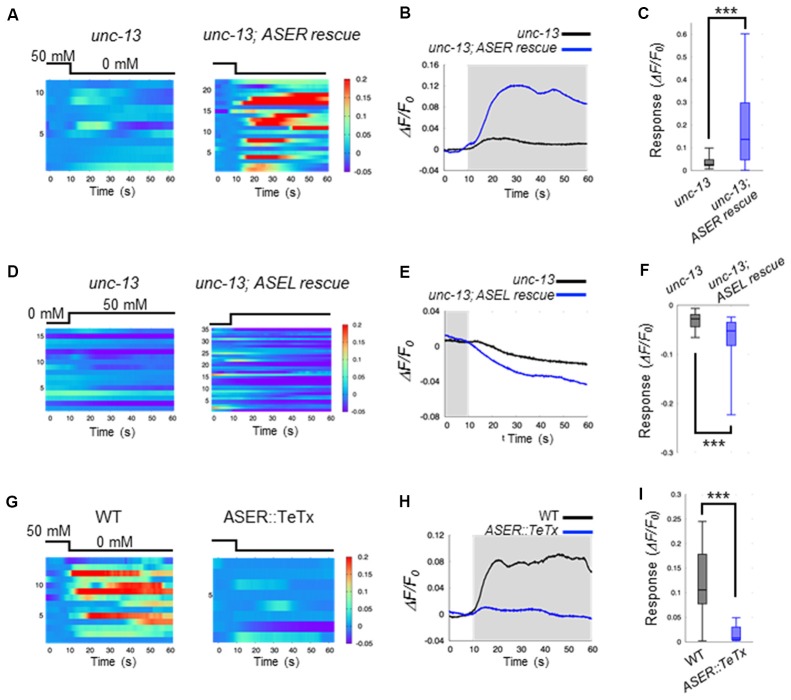
ASEL inhibits AIB whereas ASER stimulates AIB. **(A)** Heatmap traces of the AIB response to a downstep in NaCl concentration in *unc-13* mutants and ASER-specific UNC-13 rescue worms on a *unc-13* mutant background. **(B)** Averaged calcium responses in **(A)**. The shaded area indicates the period corresponding to 0 mM NaCl. **(C)** Quantitative analysis of the maximum calcium responses during a downstep in NaCl concentration in **(A)**. The error bars indicate the SEM; ***p < 0.001; Wilcoxon rank sum test. **(D)** Heatmap traces for AIB responses to an upstep in NaCl concentration in *unc-13* mutants and ASEL-specific UNC-13 rescue worms on an *unc-13* mutant background. **(E)** Averaged calcium responses in **(B)**. The shaded area indicates the period corresponding to 0 mM NaCl. **(F)** Quantitative analysis of the minimum calcium responses in AIBs during the first 10 s after an upstep in NaCl concentration. The error bars indicate the SEM; ***p < 0.001; Wilcoxon rank sum test. **(G)** Heatmap traces for the AIB responses in wild-type and transgenic worms expressing *TeTx* specifically in the ASER neuron. **(H)** Averaged calcium responses in **(G)**. **(I)** Quantitative analysis of the maximum calcium responses during a downstep in NaCl concentration in **(G)**. The error bars indicate the SEM; ***p < 0.001; Wilcoxon rank sum test.

Our results suggest that ASE neurons are the main regulators of AIB activity during salt-chemotaxis. To confirm the role of each ASE neuron in the AIB response to changes in NaCl concentration, we analyzed the neurotransmission from the ASEL or the ASER neuron to AIBs. First, we monitored AIB responses to changes in NaCl concentration in the synaptic transmission-defective mutant *unc-13* (Richmond et al., 1999). *unc-13* encodes a protein required for vesicle priming at the presynapse and mutations in this gene cause severe defects in neurotransmitter release. The AIB neurons in *unc-13(e312)* mutants did not show any response to either a downstep or upstep in NaCl concentration (Figures 2A–F). Therefore, together with our findings in the *che-1* mutant, these results suggest that synaptic transmission from ASE neurons is required to regulate AIB activity when NaCl concentrations change.

We further examined how the two distinct ASEL and ASER neuronal responses to changes in NaCl concentration cooperatively modulate AIB activity. To answer this question, we monitored AIB responses when only ASEL or ASER synaptic transmission was functional. To this aim, UNC-13 protein was specifically expressed in either ASEL or ASER neurons in the *unc-13(e312)* mutant background to recover synaptic transmission from one of these neurons. We then monitored AIB calcium responses in these cell-specific synaptic rescue worms. With regards to ASER function, we found that ASER activates AIBs only when the NaCl concentration is decreased. In worms in which transmission from the ASER was rescued by cell-specific expression of UNC-13 in the ASER neuron, we observed that the calcium response in AIB neurons increased just after the NaCl concentration is decreased (Figures 2A–C). However, a strong inactivation following an increase in NaCl concentration was not observed in this rescue animal. These results suggest that the ASER neuron may activate AIBs when NaCl decreases, but likely has no inhibitory effect on AIB activity when NaCl increases. To further confirm these results, we also monitored AIB activity in transgenic worms in which the tetanus toxin light chain from Clostridium tetani (*TeTx*) was expressed in a cell-specific manner to block synaptic transmission. *TeTx* expression reduces presynaptic vesicle release by cleaving synaptobrevin/VAMP protein, a core component required for synaptic vesicle fusion (Schiavo et al., 1992). The transgenic worms expressing *TeTx* specifically in the ASER elicited a significantly weaker calcium response in AIBs during a downstep of in NaCl concentration compared to control worms (Figures 2G–I). Therefore, we conclude that the ASER signal probably excites AIB neurons when the NaCl concentration decreases.

With regards to ASEL function, we found that ASEL signaling provides an inhibitory signal to inactivate AIB activity. AIB neurons were rapidly inactivated during an upstep in NaCl concentration, and this inactivation was also observed when the synaptic transmission from the ASEL neuron was specifically rescued upon expressing UNC-13 in the ASEL neuron of *unc-13* mutants (Figures 2D–F). Conversely, AIB activation in response to a downstep in NaCl concentration was not observed in this transgenic worm, suggesting that the ASEL may not have an excitatory role in AIB activation. Therefore, we conclude that the ASEL signal likely inactivates AIBs in response to ASEL transient activity when NaCl concentrations increase.

### Distinct Glutamatergic Signals From ASEL and ASER Neurons Affect AIB Neuronal Activity

**Figure 3:**
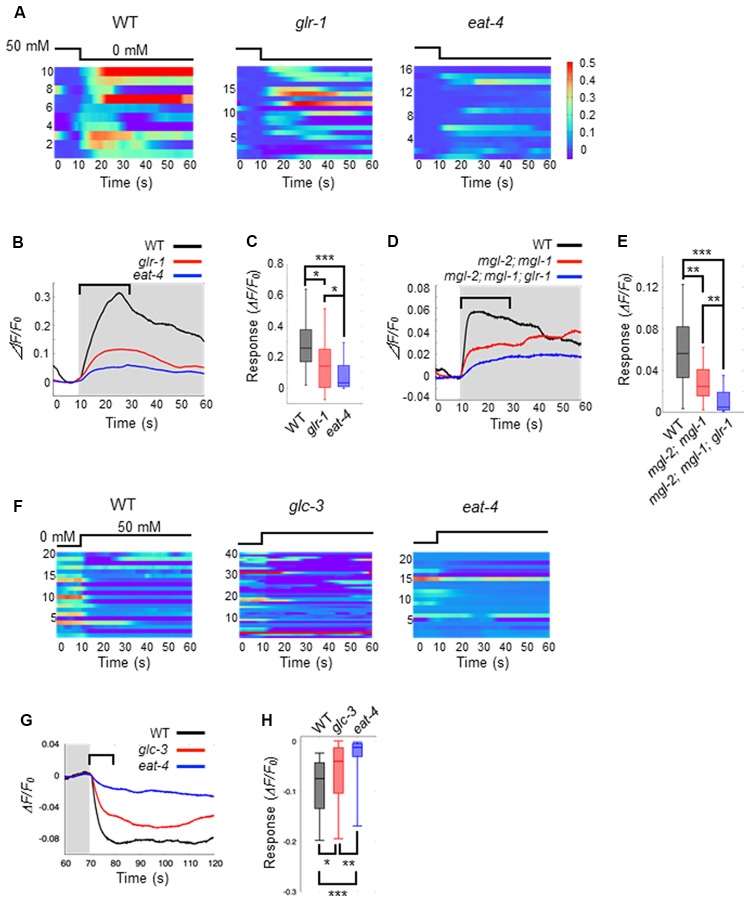
Glutamate released by ASE neurons and its receptors on AIB neurons elicit AIB excitation and inhibition. **(A)** Heatmap traces for AIB responses to a downstep in NaCl concentration in wild-type, *glr-1(n2461)* and *eat-4(ky5)* mutants. **(B)** Averaged calcium responses to a downstep in NaCl concentration in **(A)**. The shaded area indicates the period of 0 mM NaCl. A 20 s window used for analysis is shown by a blanket. **(C)** Quantitative analysis of the maximum calcium responses during 20 s of a downstep in NaCl concentration in **(A)**. The error bars indicate the SEM; *p < 0.05; ***p < 0.001; Wilcoxon rank sum test with Bonferroni correction. **(D)** Averaged calcium responses to a downstep in NaCl concentration in wild-type (black), *mgl-2(tm355); mgl-1(tm1811)* double mutants (red) and *mgl-2; mgl-1; glr-1* triple mutants (blue) expressing GCaMP6 (*n* ≧ 18). **(E)** Quantitative analysis of the maximum calcium responses during 20 s of a downstep in NaCl concentration in **(D)**. The error bars indicate the SEM; **p < 0.01, ***p < 0.001; Wilcoxon rank sum test with Bonferroni correction. **(F)** Heatmap traces for AIB responses to an upstep in NaCl concentration in wild-type, *glc-3(ok321)* and *eat-4(ky5)* mutants. **(G)** Averaged calcium responses to an upstep in NaCl concentration in **(F)**. The shaded area indicates the period of 0 mM NaCl. A 10 s window used for analysis is shown by a blanket. **(H)** Quantitative analysis of the minimum calcium responses during the 10 s of an upstep in NaCl concentration (blanket in **G**). The error bars indicate the SEM; *p < 0.05, **p < 0.01, ***p < 0.001; Wilcoxon rank sum test with Bonferroni correction.

Thus far, we have shown that ASEL and ASER activities have opposing effects on AIB activity in response to changes in NaCl concentration (Figures 1B,D). Both ASEL and ASER neurons connect to AIB neurons *via* chemical synapses and probably release glutamate as a neurotransmitter (Serrano-Saiz et al., 2013). To test how glutamate delivers both as an excitatory and inhibitory signal to AIBs, we monitored the AIB calcium response in the *eat-4(ky5)* mutant. *eat-4* encodes a vesicular glutamate transporter in *C. elegans*, and mutations in this gene cause lack or decrease of glutamate in synaptic vesicles (Lee et al., 1999). We found that *eat-4(ky5)* mutants did not show a clear AIB response to either an increase or decrease in NaCl concentration (Figure 3). These results suggest that the glutamate signal from each ASE neuron likely generates both excitation and inhibition in AIBs.

AMPA-type ionotropic glutamate receptors can act as excitatory postsynaptic receptors for odor-evoked responses in AIB neurons (Chalasani et al., 2007). As such, we questioned whether the same AMPA-type glutamate receptors also mediate AIB activity in response to changes in NaCl concentration. Here, we used *glr-1*mutants in which expression of the non-NMDA glutamate receptor is disrupted. In *glr-1(n2461)* mutants, the averaged AIB calcium response was weaker than that elicited in wild-type worms but higher than that in *eat-4* mutant worms (Figures 3A–C). Although several studies have reported that AMPA-type excitatory receptors might modulate AIB activity *via* glutamate release (Chalasani et al., 2007; Piggott et al., 2011), we conclude that both GLR-1 and other receptors coordinately contribute to AIB neuronal excitation during exposure to decreases in NaCl concentration. This lower calcium responses in AIB neurons of *glr-1* mutants was rescued by the AIB-specific expression of GLR-1 receptor, suggesting the cell-autonomous regulation of AIB neuronal activity by GLR-1 (Supplementary Figure S1). GLR-1 is not required for inactivation in response to increase of NaCl concentration because *glr-1* mutant animals showed similar calcium responses with wild-type animals upon increase of NaCl concentration (Supplementary Figure S2).

The metabotropic G-protein-coupled glutamate receptors (encoded by three *mgl* genes *mgl-1*, *mgl-2* and *mgl-3*) are thought to be expressed in AIB interneurons and act as excitatory postsynaptic receptors (Dillon et al., 2006). We next studied whether these metabotropic glutamate receptors contribute to AIB activity using *mgl-1(tm1811)*, *mgl-2(tm355)*, and their double mutant worms. Here, we found that the calcium responses in AIB neurons of the double mutants rose slowly to a peak following a downstep in NaCl concentration. On the other hand, the calcium responses in wild-type animals rose rapidly to a peak within 20 s after a downstep in NaCl concentration (Figures 3D,E). Furthermore, *glr-1*, *mgl-1* and *mgl-2* triple mutant animals showed significantly lower calcium responses than *mgl-1; mgl-2* double mutant animals after a downstep in NaCl concentration (Figures 3D,E). These results suggest that both AMPA-type and metabotropic glutamate receptors are required for rapid AIB neuronal activity after a downstep in NaCl concentration.

As for inhibitory synaptic signaling *via* glutamate, a glutamate-gated chloride ion channel (encoded by four *glc* genes and two *avr* genes in *C. elegans*) can act as an inhibitory postsynaptic receptor (Cully et al., 1994; Hart et al., 1995; Maricq et al., 1995; Yates et al., 2003; Dillon et al., 2006). Mutations in *glc-3*, a subunit of the glutamate-gated chloride ion channel, can cause decreased activity of the glutamate-gated chloride ion channel (Cully et al., 1994). We hypothesized that the ASEL neuron may inhibit AIB activity *via* this glutamate-gated chloride ion channel on AIB neurites. To test this hypothesis, we examined the calcium response in AIB neurons of *glc-3(ok321)* mutants during an increase in NaCl concentration. We found that AIB responses in *glc-3(ok321)* mutants showed a slightly slower decay in activity than the responses in wild-type animals. However, AIB activity returned to baseline faster in *glc-3(ok321)* mutants than *eat-4* mutants (where glutamate in synaptic vesicles is lost; Figures 3F–H). This slower inactivation was rescued by the AIB-specific expression of GLC-3, suggesting the cell-autonomous function of GLC-3 in AIB neurons (Supplementary Figure S1). These results suggest that the glutamate-gated chloride ion channel is required for AIB neuronal inactivation in response to an increase in NaCl concentration. The GLC-3 channel does not function for the fast excitation of AIB neurons when NaCl concentration is decreased (Supplementary Figure S2).

### The AMPA-Type Glutamate Receptor and the Glutamate-Gated Chloride Channel Are Differentially Localized on the AIB Neurite

**Figure 4:**
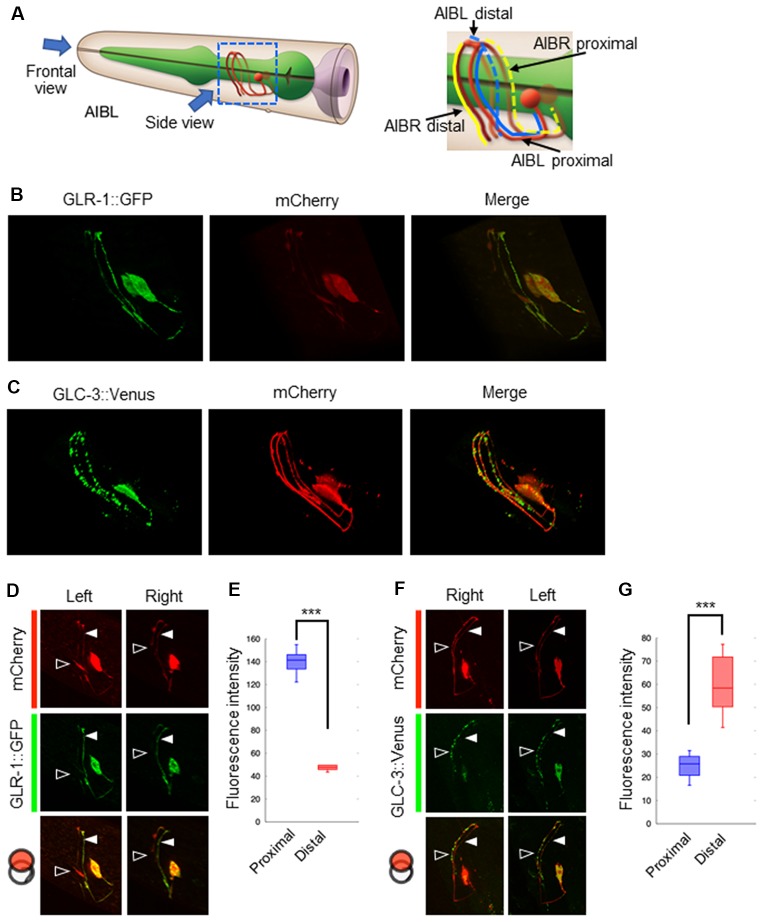
Localization patterns of the AMPA-type glutamate receptor and the glutamate-gated chloride ion channel on AIB neurites. **(A)** Left: schematic cartoon showing the morphology of AIBL neurons, modified from worm atlas (http://www.wormatlas.org). Each arrow indicates the direction for frontal-view observation or side-view observation, respectively. Right: blue and yellow lines indicate the AIBL or AIBR neurite respectively. From left-side view, both the proximal AIBL neurite and the distal AIBR neurite can be clearly observed. **(B)** The localization patterns of the AMPA-type glutamate receptor on the AIB neurites. The GFP-fused GLR-1 is expressed specifically in the AIB neurons indicated by the mCherry marker. **(C)** The localization patterns of the glutamate-gated chloride ion channel on the AIB neurites. The Venus-fused GLC-3 is expressed in the AIB neurons indicated by the mCherry marker. **(D)** One-side images for the localization of the GLR-1::GFP fusion protein in AIB neurons. Left images show the localization patterns of the fluorescent proteins in both the AIBL proximal neurite and AIBR distal neurite from each neuronal cell body. Right images show the localization patterns of the fusion proteins in both the AIBR proximal neurite and the AIBL distal neurite from each neuronal cell body. Open and closed arrowheads mark each distal or proximal neurite, respectively. **(E)** The averaged fluorescence intensity of the GLR-1::GFP fusion protein in the proximal or distal region of AIB neurons, respectively. ***p < 0.001, Wilcoxon rank sum test (*n* = 11). **(F)** One-side images for the localization patterns of the GLC-3::GFP fusion protein in AIB neurons. Right images show the localization patterns of the fluorescent proteins in both the AIBR proximal neurite and AIBL distal neurite from each neuronal cell body. Left images show the localization patterns of the fusion proteins in both the AIBL proximal neurite and the AIBR distal neurite from each neuronal cell body. Open and closed arrowheads mark each distal or proximal neurite, respectively. **(G)** The averaged fluorescence intensity of GLC-3::Venus fusion protein in the proximal or distal region of AIB neurons. ***p < 0.001, Wilcoxon rank sum test (*n* = 13).

Our results suggest that glutamate released by the ASER neuron generates an excitatory response in AIB neurons, probably *via* AMPA-type and metabotropic G-protein-coupled glutamate receptors. Conversely, glutamate released by the ASEL neuron may cause AIB inhibition *via* the glutamate-gated chloride ion channel. Because both neurons use glutamate as a transmitter, we wondered whether each synaptic site (or receptor) is randomly located on the AIB neurites or any positional arrangements exist. To answer this question, we analyzed the localization patterns of both the AMPA-type glutamate receptor GLR-1 and the glutamate-gated chloride ion channels GLC-3 on the postsynaptic AIB neurite. Because the process of each AIB neuron runs around the nerve ring from ventral to dorsal at the side of cell body and comes back to ventral at opposite side, we divided the neurite into two regions; “proximal region” which is the neurite from the cell body to dorsal midline, and “distal region” from dorsal midline to the tip of neurite (Figure 4A). In the transgenic animals expressing a GLR-1::GFP fusion protein specifically in the AIB, the GFP signal seemed to be localized uniformly across the neurite due to many small puncta (Figure 4B and see below for further observation). In the transgenic worms expressing a GLC-3::Venus in the AIB, however, the Venus signal was observed as several size of puncta (Figure 4C). Furthermore, we found that the GLR-1 fusion protein predominantly localized to the proximal region of the AIB cell body (Figures 4D,E), whereas the GLC-3 fusion protein was predominantly localized distal to the AIB cell body (Figures 4F,G). Thus, the localization patterns of GLR-1::GFP and GLC-3::Venus were strikingly different on the AIB neurite.

### Coordinated Synapse Formation Between ASE-to-AIB May Fine-Tune Postsynaptic Responses to Opposing Excitatory and Inhibitory Signals

**Figure 5:**
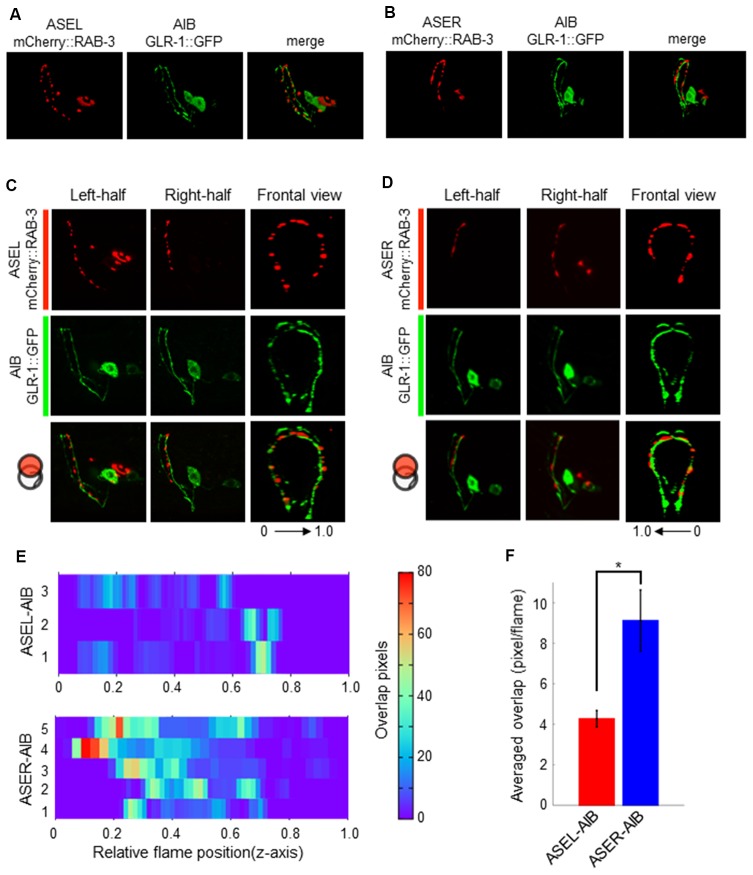
The localization patterns of the presynaptic protein RAB-3 in ASEL/R neurons and the postsynaptic AMPA-type glutamate receptors in AIB neurons. **(A)** The localization patterns of RAB-3 in the ASEL and the AMPA-type glutamate receptor GLR-1 in the AIB. **(B)** The localization patterns of RAB-3 in the ASER and the AMPA-type glutamate receptor GLR-1 in the AIB. **(C)** One-side images from left or right, and frontal images for the localization patterns of mCherry::RAB-3 on ASEL and GLR-1::GFP on the AIB. The left-half images show the co-localization pattern of mCherry::RAB-3 on the proximal ASEL and GLR-1::GFP in both the AIBL proximal neurite and AIBR distal neurite. The right side shows the co-localization pattern of mCherry::RAB-3 on the distal ASEL and GLR-1::GFP in both the AIBR proximal neurite and AIBL distal neurite. 0–1.0 shows the corresponding *Z*-axis frame position in **(E)**. **(D)** One-side images from left or right, and frontal images for the localization patterns of mCherry::RAB-3 on ASER and GLR-1::GFP on AIB. The left-side images show the co-localization pattern of mCherry::RAB-3 on the distal ASEL and GLR-1::GFP in both the AIBL proximal neurite and AIBR distal neurite. The right side shows the co-localization pattern of mCherry::RAB-3 on the proximal ASER and GLR-1::GFP in both the AIBR proximal neurite and AIBL distal neurite. 0–1.0 shows the corresponding *Z-axis* frame position in **(E)**. **(E)** Heatmap images of colocalization between mCherry (RAB-3) and GFP (GLR-1). Each colored line represents the overlap between green and red fluorescence in single slice image from Z-stack acquisition of corresponding transgenic worms. Numbers in left indicate individual animals. 0–1.0 is the relative flame position in full Z-stack image. **(F)** Quantification of overlap position in **(E)**. The number of overlapped pixels in each *z-axis* frame are averaged. The error bars indicate the SEM; *p < 0.05; Wilcoxon rank sum test.

Although the AMPA-type glutamate receptor and the glutamate-gated chloride ion channel seem to be localized at distinct regions on the AIB neurite, we could not confirm whether each receptor really localizes at distinct regions on the neurite due to the localization of many fluorescent puncta on the neurite. We also could not confirm whether those localizations really corresponded to their specific functions as inhibitory (with ASEL) or excitatory (with ASER) synapses. To investigate these questions, we first observed the co-localization of a presynaptic vesicle-associated protein, RAB-3, on the ASEL or ASER and the postsynaptic GLR-1 on the AIB (Figures 5A,B). The positions of mCherry::RAB-3 on the ASEL and GLR-1::GFP on the AIB neurite were not well-correlated with each other, only a few GFP puncta overlapped with the mCherry signal on the ASEL neurite (Figures 5C,E,F). On the other hand, the positions of that on the ASER and that on the AIB neurite closely localized each other (Figures 5D–F). Especially, many synapses of the ASER localize to the proximal region of the AIB neurite (Figure 5E). The overlap between ASER and AIB was significantly larger than that between ASEL and AIB (Figure 5F). These results suggest that the AMPA-type glutamate receptor GLR-1 on the AIB predominantly localize on the proximal neurite to form synapses with the ASER and can receive excitatory glutamate signals from it.

**Figure 6:**
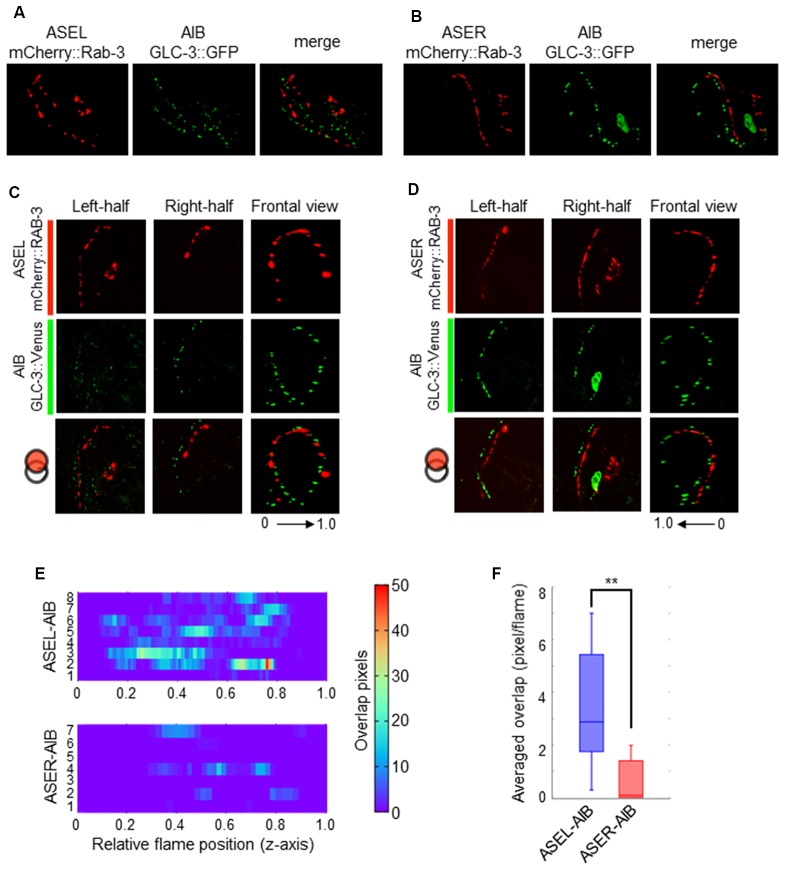
The localization patterns of the presynaptic protein RAB-3 in ASEL/R neurons and the postsynaptic glutamate-gated chloride ion channel in AIB neurons. **(A)** The localization patterns of RAB-3 in the ASEL and the glutamate-gated chloride channel GLC-3 in the AIB. **(B)** The localization patterns of RAB-3 in the ASER and the glutamate-gated chloride channel GLC-3 in the AIB. **(C)** One-side images from left or right, and frontal images for the localization patterns of mCherry::RAB-3 on ASEL and GLC-3::Venus on the AIB. The left-half images show the co-localization pattern of mCherry::RAB-3 on the proximal ASEL and GLC-3::Venus in both the AIBL proximal neurite and AIBR distal neurite. The right side shows the co-localization pattern of mCherry::RAB-3 on the distal ASEL and GLC-3::Venus in both the AIBR proximal neurite and AIBL distal neurite. **(D)** One-side images from left or right, and frontal images for the localization patterns of mCherry::RAB-3 on ASER and GLC-3::Venus on the AIB. The left-half images show the co-localization pattern of mCherry::RAB-3 on the distal ASER and GLC-3::Venus in both the AIBL proximal neurite and AIBR distal neurite. The right side shows the co-localization pattern of mCherry::RAB-3 on the proximal ASER and GLC-3::Venus in both the AIBR proximal neurite and AIBL distal neurite. **(E)** Heatmap images of colocalization between mCherry (RAB-3) and GFP (GLR-1). Each colored line represents the overlap between green and red fluorescence in single slice image from Z-stack acquisition of corresponding transgenic worms. Numbers in left indicate individual animals. 0–1.0 is the relative flame position in full Z-stack image. **(F)** Quantification of overlap between RAB-3 on the ASEL or ASER and GLC-3 on the AIBs. The number of overlapped pixels in each frame are averaged. The error bars indicate the SEM; **p < 0.01; Wilcoxon rank sum test.

Contrary to the ASER-to-AIB excitatory signal, our calcium imaging data showed that ASEL glutamatergic signaling inhibits AIB activity (Figure 3). We thus hypothesized that the ASEL and AIB may form inhibitory synapses, and that both the presynaptic and postsynaptic components of the synapse can closely localize with each other. To prove this, we observed the localization pattern of mCherry::RAB-3 on ASEL and ASER neurites and GLC-3::Venus on AIB neurites. We found that the puncta of mCherry::RAB-3 on the ASEs were mainly facing the proximal region of the AIB neurons whereas the puncta of GLC-3::Venus were localized strongly on the distal region of AIB neurons (Figures 4G, 6A,B). These results suggest that the presynaptic sites labeled by mCherry::RAB-3 on ASE neurons and postsynaptic GLC-3 accumulation on AIB neurons are distantly located, compared to the sites of RAB-3 on the ASE neurons and postsynaptic GLR-1 on the AIB (Figures 5A,B). So, we found that only a small number of mCherry::RAB-3 puncta on the ASEL was closely aligned with the GLC-3::Venus on the AIB (Figures 6C,E). On the other hand, mCherry::RAB-3 on the ASER was distinct from the distribution of GLC-3::Venus on the AIB, few overlap was observed (Figures 6D,E). The averaged overlap between ASEL and AIB was significantly larger than those between ASER and AIB (Figures 6E,F). These results suggest that a few but clear synapses are formed between the ASEL and AIB, and at those synapses, the glutamate-gated chloride ion channel GLC-3 on the AIB receives inhibitory glutamate signals from the ASEL.

## Discussion

In this study, we show that the excitatory/inhibitory switch from asymmetric sensory neurons defines smooth and fine transitions of responses to changes in salt concentration in *C. elegans* postsynaptic neurons. Previous studies have indicated that the ASER neuron stimulates AIBs (Kunitomo et al., 2013), whereas the ASEL neuron inhibits AIBs (Wang et al., 2017), though molecular components in these neuronal regulations has not been revealed. Our results on excitatory/inhibitory signaling patterns are consistent with these previous studies. Based on the expression of EAT-4 vesicular glutamate transporter in these two neurons, it is highly possible that these neurons release glutamate as transmitter. By using several presynaptic and postsynaptic mutants, we confirmed that glutamate from the ASEL neuron is really required to inhibit AIBs *via* a glutamate-gated chloride channel, whereas glutamate from the ASER neuron activates AIBs *via* two types of glutamate receptors. Furthermore, we found a differential distribution of each excitatory or inhibitory receptor on AIB neurites: excitatory synapses are mainly located proximal to the AIB cell body but inhibitory synapses are distal to the AIB cell body. These results suggest that the two glutamatergic signals govern fine, single-cell activity in response to environmental stimuli, and that a unique mechanism for excitatory/inhibitory transformation exists for circuit dynamics in animal behavioral strategy.

For ASER-to-AIB signaling, we show that glutamate and its receptors mediate activation of AIB neurons during a downstep in NaCl concentration. Several glutamate receptors are expressed in AIB neurons. For example, GLR-1 receptors operate as an excitatory receptor during odor-evoked behaviors, and *glr-1* mutants show a lack of AIB excitation by odor stimulation and defective odor-evoked chemotaxis (Chalasani et al., 2007). As such, we suspected that GLR-1 might also function as an excitatory receptor to receive glutamate from the ASER during decreases in NaCl concentration (Figure 3). The calcium response in the AIB neurons of *glr-1* mutants was decreased compared to wild-type, but not completely lost as observed in *eat-4* mutant animals. Supporting our results, the salt-chemotaxis behavior of *glr-1* mutants is comparable to that of wild-type animals (Kano et al., 2008). Thus, the contribution of the GLR-1 receptor in AIB excitation is not sufficient for mediating salt-chemotaxis behavior. The AMPA-type glutamate receptor functions in a heteromeric complex with several GLR subunits such as GLR-1/GLR-2 in osmotic avoidance response (Mellem et al., 2002); disrupting the single AMPA-type glutamate receptor subunit might not be effective in abolishing the glutamatergic signal for the salt-chemotaxis. Furthermore, other glutamate-gated cation channels (except for AMPA-type glutamate receptors) may also affect AIB excitation. Consistent with this hypothesis, AIB responses in *mgl-2; mgl-1* double mutants decreased the initial activation of response during the decrease in NaCl concentration, and triple mutants with *glr-1* further decreased its activation (Figures 3D,E). Thus, the metabotropic glutamate receptors probably contribute to initial AIB activity in initial phase of detecting a downstep in NaCl. Further analyses will reveal the exact postsynaptic components for the excitatory synapses in AIBs.

We also showed that activated AIBs are rapidly inactivated when NaCl concentration increases, suggesting the receipt of an inhibitory signal at this point. Four *glc* genes and two *avr* genes in the *C. elegans* genome are associated with glutamate-evoked inactivation of neuronal cells. Mutations in the *avr-14* gene cause a lack or decrease in glutamate-evoked current in AIB neurons (Summers et al., 2015). In this study, we showed that a mutation in *glc-3* causes weaker inhibition of AIBs during increases in NaCl than that of wild-type animals, suggesting that at least GLC-3 is involved in the inhibitory synaptic transmission between ASEL and AIBs. However, *eat-4* mutant animals showed even weaker inhibition of AIBs, therefore, suggesting that several other glutamate-gated chloride ion channels might be expressed on AIBs and function as inhibitory receptors.

Interestingly, we showed that AIB neurons are weakly activated by a downstep in NaCl concentration, even in *eat-4* mutants (Figure 3). This weak activation was not observed in *che-1* mutants that lack ASE sensory activity (Figure 1). On the other hand, a previous study reported that the AIB response to odor stimulation, which is mainly received by AWC sensory neurons and transmitted to AIB neurons, was almost fully lost in *eat-4* mutants (Chalasani et al., 2007). Based on this discrepancy, we hypothesize that not only glutamate but also other signals from ASEs may affect AIB activity. A neuropeptide may be a candidate signaling molecule because ASEs release an insulin-like peptide, and this peptide can activate surrounding neurons expressing DAF-2 receptors (Leinwand and Chalasani, 2013). AIB neurons may also be regulated by this peptidic signaling, either directly or indirectly. Furthermore, non-glutamatergic signaling may also downregulate AIB activity upon increases in NaCl concentration. Compared to a lack of response in *che-1* mutants, slight inactivation was observed in *eat-4* mutants (compare Figures 1C, 3G). The ASEL neuron releases insulin during increases in NaCl concentration, suggesting that the same peptide may be used to inactivate AIB (Leinwand and Chalasani, 2013).

We also examined the relationship between synapse formation on AIBs and its significance for their activities. On the AIB neurons, the proximal neurites receive synaptic inputs from both sensory and upper-layer interneurons, whereas the distal neurites receive synaptic inputs from lower-layer interneurons and motor neurons (White et al., 1986). This clear segregation of sites of synaptic input is likely associated with the fine regulation of neuronal activity. A previous study reported that each type of glutamate receptor shows a different distribution pattern along the AIB neurite (Summers et al., 2015). Consistent with this result, we found that the localization patterns of GLR-1 and those of GLC-3 are strikingly different on the AIB neurite: GLR-1 receptors are localized predominantly at the proximal region, whereas GLC-3 receptors are localized at the distal region. These distinct localization patterns of excitatory and inhibitory receptors in one neurite is likely important for AIB activity and function. Although we have not determined the exact role for excitatory and inhibitory signaling in AIB tuning, a lack in inhibitory signal from the ASEL neuron abolished the rapid AIB inactivation when NaCl concentration increased. We believe that this rapid inactivation is probably required to sense subtle changes in sensory signals and to control precise chemotaxis behaviors to different salt concentrations. As well as salt-chemotaxis neurons ASE, other sensory neurons such as ASH and AWC form chemical synapses at the proximal region of the AIB neurite (White et al., 1986), and these synapses stimulate AIBs *via* EAT-4-dependent glutamatergic signaling (Chalasani et al., 2007; Piggott et al., 2011). To understand the meaning of these unbalanced synaptic inputs on a specific postsynaptic target cell, further experiments that monitor developmental stage-specific synapse formation and/or elimination between these sensory neurons and AIB neurons, are required. We believe that these analyses may answer how a sensory-evoked AIB excitation might govern reversal behavior in the worm, whereas feedback from interneurons and motor neurons for AIB inhibition might promote forward movement. We also believe that our study provides a framework for sensorimotor integration system at cellular resolution.

Information processing in the *C. elegans* salt-chemotaxis circuit is similar to the thermotaxis circuit that is composed of the AFD and AWC thermosensory neurons and AIY interneurons. The AFD-glutamatergic signal strongly inhibits AIY activity *via* GLC-3 receptors. By contrast, the AWC-glutamatergic signal weakly activates AIY *via* unknown receptors (Ohnishi et al., 2011). In addition, AWC neurons, which can sense both temperature and odor, regulate the activity of two classes of postsynaptic interneurons *via* glutamatergic signaling. This AWC signal inhibits AIY *via* GLC-3 inhibitory glutamate receptors, whereas activates AIBs *via* GLR-1 excitatory glutamate receptors (Chalasani et al., 2007). The same glutamate neurotransmitter from AWC sensory neurons is used to regulate distinct types of postsynaptic glutamate receptors in different classes of interneurons. However, the significance of this neural mechanism by which the same synaptic transmitter from one presynaptic neuron can generate an opposite postsynaptic neuronal-state is still poorly understood (Ohnishi et al., 2011; Wang et al., 2017). Here we provide a similar but more compact neural signaling mechanism, which may be important in regulating opposing behavioral states.

Our data suggest that the activity of individual neurons can be finely tuned by dynamic synaptic inputs by one neurotransmitter, which is transformed into either an excitatory or inhibitory signal *via* distinct receptors in single postsynaptic cells. This simple but well-organized neural mechanism may be converged to achieve numerous behavioral outcomes using only a limited number of neuronal cells in *C. elegans*. By employing these mechanisms, *C. elegans* can detect complex and dynamic environmental changes by its sensory system, and this paradigm can quickly enable the production of a suitable behavior. The identification of these converged neuronal mechanisms in *C. elegans*, from sensory neurons to first-layer interneurons, provides a novel insight into the more complex information processing in other neural circuits. Further studies on the ASEs circuits in *C. elegans* are now warranted to improve our understanding of the relationship between sensory inputs and synaptic regulation for fine-tuned responses in postsynaptic neurons, such as AIBs.

## Author Contributions

MK and MD designed the experiments and wrote the article. MK performed all the experiments and analyzed the data.

## Conflict of Interest Statement

The authors declare that the research was conducted in the absence of any commercial or financial relationships that could be construed as a potential conflict of interest. The reviewer KK and handling editor declared their shared affiliation at time of review.
