# Supplementary material for: An Excitatory/Inhibitory Switch From Asymmetric Sensory Neurons Defines Postsynaptic Tuning for a Rapid Response to NaCl in Caenorhabditis elegans
Source: Front Mol Neurosci. 2019 Jan 9;11:484. doi: 10.3389/fnmol.2018.00484 (PMC6333676; doi:10.3389/fnmol.2018.00484)
Supplement: Supplementary file 1 [file Table_1.docx]

**Supplementary figure 1. The cell-autonomous regulation of AIB neuronal responses by GLR-1 and GLC-3.**

1. Averaged calcium responses of AIB neurons to a downstep in NaCl concentration in *glr-1* mutants and AIB-specific GLR-1 rescue worms on a *glr-1* mutant background. The shaded area indicates the period corresponding to 0 mM NaCl, and a 20 s window used for quantitative analyses in (B) is shown by a blanket.
2. Quantitative analysis of the maximum calcium responses during 20 s of a downstep in NaCl concentration in A. Boxes show the median and first and third quartiles; **p < 0.01; Wilcoxon rank sum test. N > 10.
3. Averaged calcium responses of AIB neurons to a upstep in NaCl concentration in *glc-3* mutants and AIB-specific GLC-3 rescue worms on a *glc-3* mutant background. The shaded area indicates the period corresponding to 0 mM NaCl, and a 10 s window used for quantitative analyses in (D) is shown by a blanket.
4. Quantitative analysis of the maximum calcium responses during 10 s of a upstep in NaCl concentration in C. Boxes show the median and first and third quartiles; *p < 0.05; Wilcoxon rank sum test. N > 16.

**Supplementary figure 2. GLR-1 is not required for the inactivation of AIB and GLC-3 is not involved in the excitation of AIB.**

1. Averaged calcium responses of AIB neurons to a upstep in NaCl concentration in *glr-1* mutants. The shaded area indicates the period corresponding to 0 mM NaCl, and a 10 s window used for quantitative analyses in (B) is shown by a blanket. A mutation in *glr-1* does not affect the inactivation of AIB activity after a upstep of NaCl concentration.
2. Quantitative analysis of the maximum calcium responses during 10 s of a upstep in NaCl concentration in A. Boxes show the median and first and third quartiles; ns, not significant; Wilcoxon rank sum test. N > 16.
3. Averaged calcium responses of AIB neurons to a downstep in NaCl concentration in *glc-3* mutants. The shaded area indicates the period corresponding to 0 mM NaCl, and a 20 s window used for quantitative analyses in (D) is shown by a blanket. The glc-3 mutant animals can show intact AIB activities in response to a downstep of NaCl concentration.
4. Quantitative analysis of the maximum calcium responses during 20 s of a upstep in NaCl concentration in (C). Boxes show the median and first and third quartiles; ns, not significant; Wilcoxon rank sum test. N = 20
