# Supplementary material for: An Excitatory/Inhibitory Switch From Asymmetric Sensory Neurons Defines Postsynaptic Tuning for a Rapid Response to NaCl in Caenorhabditis elegans
Source: Front Mol Neurosci. 2019 Jan 9;11:484. doi: 10.3389/fnmol.2018.00484 (PMC6333676; doi:10.3389/fnmol.2018.00484)
Supplement: Supplementary file 2 [file Table_2.DOCX]

**Supplementary table 1. Strain list**

| DK5349 | *taEx138[Pgcy-5::G-GECO1.2, Plin-44::mCherry]* | 1B, 1C |
| --- | --- | --- |
| DK5366 | *taEx154[Pgcy-7::G-GECO1.2,Plin-44::mCherry]* | 1B, 1C |
| DK5389 | *taEx171 [Pnpr-9::GCaMP6, Plin-44::mCherry]* | 3D, 3E |
| DK5393 | *taEx175[Pnpr-9::G-GECO1.2, Plin-44::mCherry]* | 1B-1E, 2G-2H,  3A-3H |
| DK5400 | *unc-13(e312)I;*  *taEx175[Pnpr-9::G-GECO1.2, Plin-44::mCherry]* | 2A-2F |
| DK5426 | *che-1(p679)I; taEx194[Pnpr-9::G-GECO1.2, Pgcy-5::mCherry, Punc-25::mCherry]* | 1B-1E |
| DK5428 | *lin-15(n765ts)X; taEx196[Pnpr-9::G-GECO1.2, Pgcy-5::TeTx::mCherry, pbLH98]* | 2G-2I |
| DK5433 | *glr-1(n2461)III;*  *taEx201[Pnpr-9::G-GECO1.2, Plin-44::mCherry]* | 3A-3C |
| DK5445 | *eat-4(ky5)III;*  *taEx209[Pnpr-9::G-GECO1.2, Plin-44::mCherry]* | 3A-3H |
| DK5463 | *unc-13(e312)I;*  *taEx226[Pgcy-5::UNC-13S::mCherry, Punc-25::mCherry];*  *taEx175 [Pnpr-9::G-GECO1.2, Plin-44::mCherry]* | 2A-2C |
| DK5465 | *glc-3(ok321)V;*  *taEx228[Pnpr-9::G-GECO1.2, Plin-44::tdTomato]* | 3F-3H |
| DK5466 | *mgl-2(tm355)I; mgl-1(tm1811)X;*  *taEx229[Pnpr-9::GCaMP6, Plin-44::mCherry]* | 3D, 3E |
| DK5477 | *lin-15(n765ts)X;*  *taEx234[Pgcy-7::mCherry::RAB-3, pbLH98];*  *taEx236[Pnpr-9::GLR-1::GFP, pbLH98]* | 5A-5F |
| DK5478 | *lin-15(n765ts)X;*  *taEx233[Pgcy-5::mCherry::RAB-3, pbLH98];*  *taEx199[Pnpr-9::GLC-3::Venus]* | 6A-6F |
| DK5479 | *lin-15(n765ts)X;*  *taEx233[Pgcy-5::mCherry::RAB-3, pbLH98];*  *taEx236[Pnpr-9::GLR-1::GFP, pbLH98]* | 5A-5F |
| DK5480 | *unc-13(e312)I; lin-15(n765ts)X;*  *taEx225[Pgcy-7::UNC-13S::mCherry, pbLH98];*  *taEx175[Pnpr-9::G-GECO1.2, Plin-44::mCherry]* | 2D-2F |
| DK5481 | *lin-15(n765ts)X;*  *taEx234 [Pgcy-7::mCherry::RAB-3, pbLH98];*  *taEx199 [Pnpr-9::GLC-3::Venus, pbLH98]* | 6A-6F |
| DK5482 | *lin-15(n765ts)X;*  *taEx235[Pnpr-9::mCherry, pbLH98];*  *taEx236 [Pnpr-9::GLR-1::GFP, pbLH98]* | 4A-4G |
| DK5483 | *lin-15(n765ts)X;*  *taEx235 [Pnpr-9::mCherry, pbLH98];*  *taEx199 [Pnpr-9::GLC-3::Venus, pbLH98]* | 4A-4G |
| DK5533 | *mgl-2(tm355)I; mgl-1(tm1811)X; glr-1(n2461) III;*  *taEx229[Pnpr-9::GCaMP6, Plin-44::mCherry]* | 3D, 3E |
| DK5523 | *glr-1(n2461)III;*  *taEx265[Pnpr-9::GLR-1, Pnpr-9::G-GECO1.2, Plin-44::mCherry]* | S1A, S1B |
| DK5524 | *glc-3(ok321)V;*  *taEx264[Pnpr-9::GLC-3, Pnpr-9::G-GECO1.2, Plin-44::tdTomato]* | S1C, S1D |
