# Supplementary figures and images for: An Excitatory/Inhibitory Switch From Asymmetric Sensory Neurons Defines Postsynaptic Tuning for a Rapid Response to NaCl in Caenorhabditis elegans
Source: Front Mol Neurosci. 2019 Jan 9;11:484. doi: 10.3389/fnmol.2018.00484 (PMC6333676; doi:10.3389/fnmol.2018.00484)

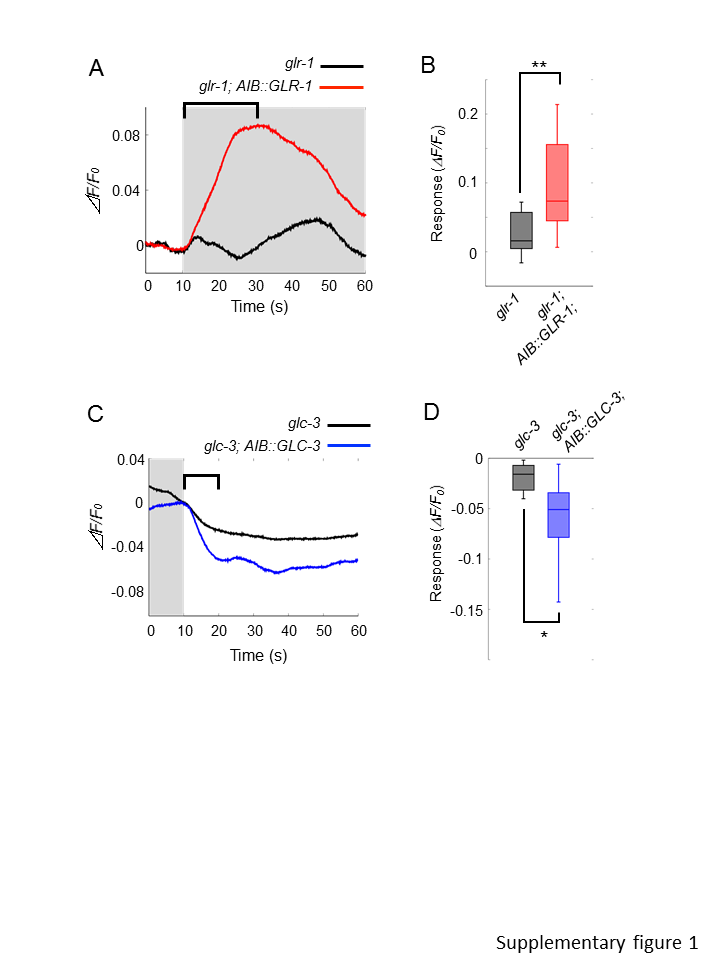

Supplement: Supplementary file 3 [file Image_1.TIF]

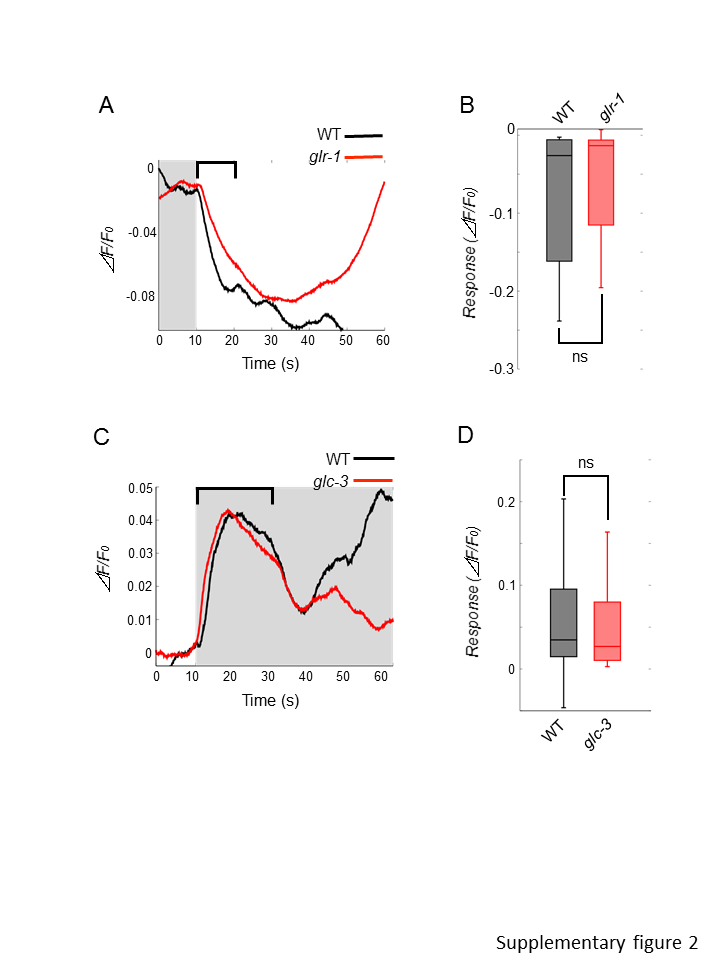

Supplement: Supplementary file 4 [file Image_2.TIF]
